# The Antioxidant Capability of Higenamine: Insights from Theory

**DOI:** 10.3390/antiox9050358

**Published:** 2020-04-25

**Authors:** Isabella Romeo, Angela Parise, Annia Galano, Nino Russo, Juan Raúl Alvarez-Idaboy, Tiziana Marino

**Affiliations:** 1 Dipartimento di Chimica e Tecnologie Chimiche, Università della Calabria, 87036 Arcavacata di Rende, Italy; isabella.romeo@unical.it (I.R.); angela.parise@unical.it (A.P.); 2 Departamento de Química, Universidad Autónoma Metropolitana-Iztapalapa, Ciudad de México 09340, Mexico; annia.galano@gmail.com; 3 Facultad de Química, Departamento de Física y Química Teórica, Universidad Nacional Autónoma de México, Ciudad de Mexico 04510, Mexico; jidaboy@unam.mx

**Keywords:** DFT, antioxidant mechanism, kinetic constants, higenamine, acid–base equilibria

## Abstract

Density functional theory was employed to highlight the antioxidant working mechanism of higenamine in aqueous and lipid-like environments. Different reaction mechanisms were considered for the reaction of higenamine with the ^•^OOH radical. The pH values and the molar fraction at physiological pH were determined in aqueous solution. The results show that the preferred reaction mechanism was the hydrogen atom transfer from the catecholic ring. The computed kinetic constants revealed that, in order to obtain reliable results, it is important to consider all the species present in water solution derived from acid–base equilibria. From the present investigation, it emerges that at physiological pH (7.4), the scavenging activity of higenamine against the ^•^OOH radical is higher than that of Trolox, chosen as a reference antioxidant. Furthermore, higenamine results to be more efficient for that purpose than melatonin and caffeine, whose protective action against oxidative stress is frequently associated with their reactive oxygen species (ROS) scavenging activity.

## 1. Introduction

**Figure 1:**
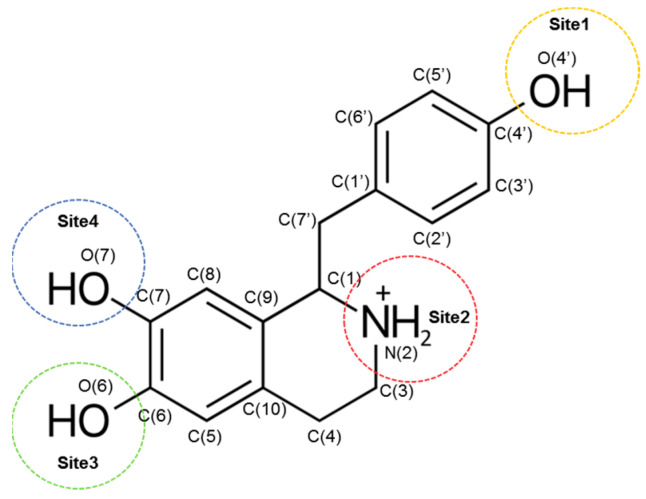
The 2D structure of higenamine.

Higenamine [1-(4′-hydroxybenzyl)−6,7-dihydroxy-1,2,3,4-tetrahydroisoquinoline] (Figure 1), also known as norcoclaurine or dl-demethylcoclaurine, is a plant-based alkaloid belonging to the structural class of protoberberines. It is present in many plants such as *Aconitum japonicum*, *Nandina Domestica*, *Gnetum Parvifolium*, *Asarum heterotropoides*, *Nelumbo nucifera*, *Galium divaricatum, Annona squamosal*, and *Aconitum carmichaelii* [1,2].

For 40 years, higenamine has been known as a cardiotonic due to its beta-agonist activity and inotropic and chronotropic properties [3]. More recently, a revived interest in this compound has been motivated by its possible applications in many other therapeutic fields. Indeed, several studies have highlighted that higenamine exerts a hypotensive effect (it is a α1-adrenergic receptor antagonist) [4] and a protective effect on ischemia/reperfusion injuries (it activates the Phosphatidylinositol-3-kinase–Protein kinase B also known as Akt (PI3K/AKT) pathway) [5] and, more recently, it has been proposed as pharmacological stress agent for myocardial perfusion imaging. In addition to these properties, higenamine exhibits pharmacological activity towards other diseases, such as sepsis, heart failure, breathing difficulties, erectile dysfunction (ED), bradyarrhythmia, arthritis, and disseminated intravascular coagulation [1]. This multi-target activity led researchers to focus on the mechanisms and on the pathways implicated in higenamine action in different diseases. Recent literature [6,7,8,9,10] indicates that the common denominator could be its antioxidant activity against reactive oxygen species (ROS). These highly reactive species may originate endogenously and exogenously, such as from metabolic pathways and external influences (e.g., smoking, radiation, drugs, and other environmental contaminants). Although under normal conditions the organism is able to maintain a good balance between production and removal of free radicals, their overproduction leads to oxidative stress, necrosis, apoptosis, and damage to biological macromolecules and compromises homeostasis and cellular function [11,12,13].

Antioxidant compounds act by detoxifying and reducing ROS and controlling oxidative stress through different reaction mechanisms, such as electron transfer (ET), proton transfer (PT), sequential proton loss electron transfer (SPLET), hydrogen atom transfer (HAT) and radical adduct formation (RAF). Considering that natural products offer a wide range of antioxidant compounds, the identification of the specific role of each chemical portion of these compounds and the associated reaction mechanism is paramount in order to overcome oxidative stress in a targeted manner [14,15].

The presence of an OH group with phenolic nature proves to be essential for these properties, particularly, in the alkoxyl, carboxyl, ester, and carbonyl groups in which the O atom provides acidity or neutrality. Uncommonly, alkaline phenolic compounds are studied for their antioxidant activity [16,17,18]. 

N-containing compounds, such as alkaloids, are abundantly present in natural products. It has been shown that these alkaloids exhibit protective action against free radicals due to the elimination of the cation radical 2,2′-azinobis-(3-ethylbenzthiazoline-6-sulfonic acid) (ABTS^•+^) [19,20] as well as to their scavenging activity against the 1,1-diphenyl-2-picryl-hydrazyl (^•^DPPH) radical and their inhibitory effects on hydrogen peroxide radical [21]. 

Recently, among the phenolic alkaloids, Xie et al. [22] studied higenamine as an attractive scaffold to test its antioxidant effect. In particular, as shown in Figure 1, the peculiarity of higenamine structure is the presence of a protonated N-atom (site 2) which provides it with a strong electron-withdrawing capacity, thus resulting in an electron density change and suggesting an effect of pH on the antioxidant power of higenamine. For all these reasons and in order to rationalize the existing experimental results [22], we decided to carefully investigate the antioxidant properties of higenamine by employing a density functional theory (DFT)-based computational protocol previously and successfully used for a series of natural antioxidants [23,24,25]. The hydroperoxyl radical (^•^OOH) was chosen because its half-life allows the best interception by chemical scavengers [26,27]. Several reaction mechanisms (HAT, single-electron transfer (SET), and RAF) were considered, and the overall kinetic behaviour was evaluated. 

## 2. Materials and Methods

All the calculations were carried out with the Gaussian 09 package of programs [28]. Full geometry optimizations and frequency calculations were done by using the DFT. The M06-2X functional coupled with the extended 6-311+G(d) basis set was chosen because of the good performance of this level of theory for kinetic calculations [29] and was successfully used in previous works for modelling chemical reactions between antioxidant species and free radicals [30,31,32,33,34,35,36]. Unrestricted calculations were used for open-shell systems. The solvent effects, in water and pentylethanoate (PE) environments, were taken into account by using the Solvent Model based on Density SMD [37], which has been proven to estimate the solvation free energies for charged or uncharged solutes with relatively low errors. Local minima and transition states (TS) were identified by the number of imaginary frequencies (0 or 1, respectively). Intrinsic reaction coordinate calculations (IRC) were performed to verify if the located TS properly connected the relative minima along the reaction coordinate [38]. Thermodynamic corrections at 298.15 K were included in the calculation of relative energies. The used computational protocol is in line with the quantum mechanics-based test for overall free-radical scavenging activity (QM-ORSA), [39,40] which was validated by comparison with experimental results. Spin density computations were performed for the most stable open-shell species. Natural bond orbital (NBO) analysis [41,42,43], as implemented in the Gaussian 09 package, was to evaluate net charges, bond order, and conjugation. 

## 3. Results and Discussion

**Figure 2:**
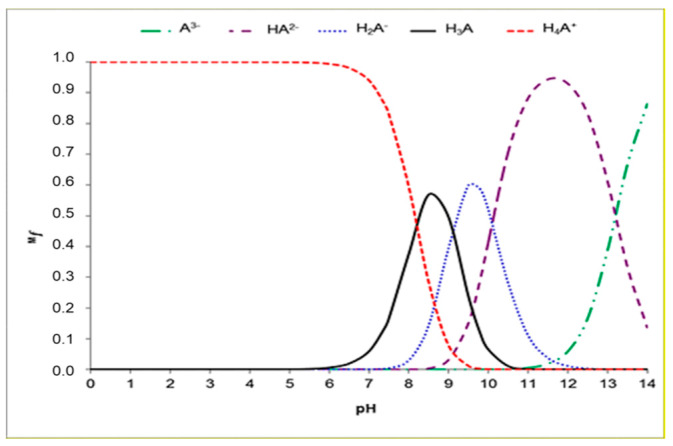
Distribution diagram of higenamine as a function of pH.

**Figure 3:**
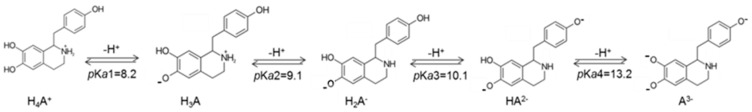
pKa values of the relative deprotonation paths of higenamine at physiological pH.

As it is well known, in the aqueous phase, knowledge of the acid−base equilibrium is crucial for the individuation of the chemical species present in physiological conditions. For this reason, our preliminary calculations were devoted to the computation of the acid dissociation constants (pKas) of the investigated compound, using the parameter fitting method [44], and to the quantification of the relative molar fractions at pH 7.4 (see Figure 2). Considering all the possible deprotonation paths (see Figure 3), our results indicated that the first deprotonation occurred at the OH in position C6 (Figure 1), and the relative pKa1 value was 8.2, in agreement with experimental results [45]. The second deprotonation at pKa2 = 9.1 (see Figure 3) involved the NH_2_ group, giving rise to the H_2_A^−^ species. Furthermore, the loss of H^+^ from the OH in position C4 generated the HA^2−^ anion (pKa3 = 10.1), while the last deprotonation (pKa4 = 13.2) generated the A^3−^ species. Looking at Table 1, it is possible to evidence that at physiological pH, in addition to the dominant H_4_A^+^ species, also the H_3_A and H_2_A^−^ species are present in aqueous solution, in molar fractions of 0.136 and 0.002, respectively.

These results indicated that for higenamine, it is necessary to take into account the species produced by the first two acid–base equilibria for the determination of the antioxidant power of higenamine toward the ^•^OOH radical in an aqueous environment.

**Scheme 1:**
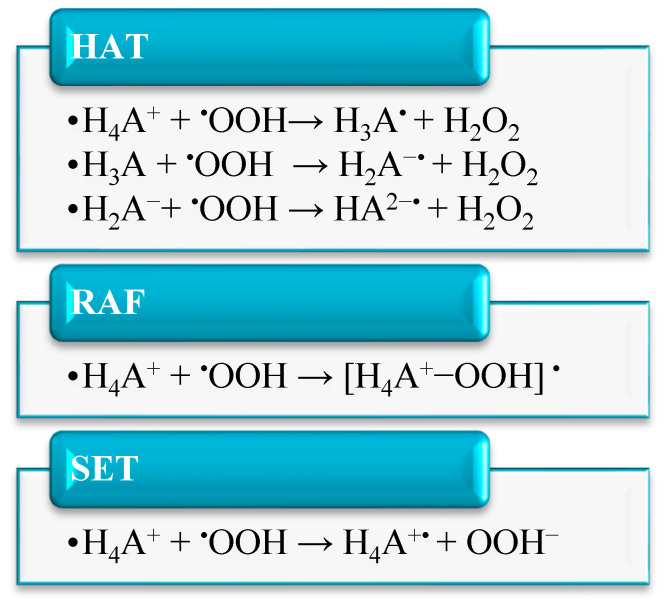
Schematic representation of the considered mechanisms for higenamine and its acid–base forms. Gibbs free energies of reaction (ΔG) and activation (ΔG^⧧^), for all populated species in aqueous and pentylethanoate (PE) solvents, involved in the studied mechanisms are summarized in Table 2. HAT, hydrogen atom transfer, RAF, radical adduct formation, SET, single-electron transfer.

The investigated reaction mechanisms are summarized in Scheme 1:

Concerning the HAT mechanism, in the H_4_A^+^ form, only the ^•^OOH attack on hydrogens at site 3 (O6) and site 4 (O7) of the catechol moiety results to be exergonic by 3.62 and 2.58 kcal mol^−1^, respectively, while at the H_3_A sites, 1 (O4′) and 4 (O7) are thermodynamically favoured. In the H_2_A^−^ species, the Gibbs energies for the reaction at sites 1 and 4 are −3.81 and −15.63 kcal mol^−1^, respectively. In addition, for the catecholic moiety in higenamine going from the neutral form to the anionic one, the trend of the obtained ΔG values well reproduces that observed for catechol [46].

The most exergonic reaction path is HAT from site 4 of the catechol moiety, for both H_2_A^−^ and H_3_A species.

**Figure 4:**
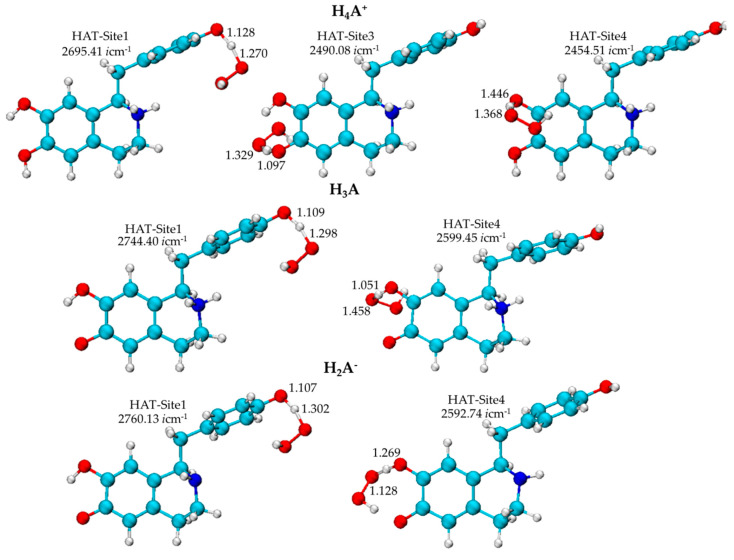
Geometries of the transition states of the H_4_A^+^, H_3_A, and H_2_A^−^ forms obtained in water as a result of hydrogen atom transfer mechanism at the M062X/6-311+G(d) level of theory.

The optimized geometries of the transition states are illustrated in Figure 4.

From Table 2, it is possible to note that the activation energy for the reaction of H_4_A^+^ species for which the TS have been characterized, assumes values ranging from about 20 to 28 kcal mol^−1^, whereas for the reaction of H_3_A and H_2_A^−^ species, the ΔG⧧ values range from 1 to 21 kcal mol^−1^.

In pentylethanoate solvent, that mimics the lipid environment and in which only the H_4_A^+^ form is present, the obtained ΔG for the ^•^OOH attack at the different sites had positive values (0.50, 6.43, 0.58 kcal mol^−1^ for O4′, O6, O7, respectively), indicating that the HAT process can hardly take place.

Concerning the RAF mechanism for the most abundant species H_4_A^+^, all ^•^OOH addition channels were found to be endergonic (Table 2), with ΔG values larger than 20 kcal mol^−1^. Generally, the kinetic calculations for endergonic channels are excluded because, although they might occur at significant rates, the reaction is reversible, and no products are observed. Instead, if the products are able to further react quickly producing a driving force and barriers are small, these processes also need to be considered [44]. For this reason, they were considered in our kinetic computation.

In a water environment, SET reactions for all the investigated species, with the exception of the anionic form, gave also endergonic ΔG (see Table 2). The degree of deprotonation contributed to increasing the thermochemical viability of the SET process, thus pointing out the role of all the existing species at physiological pH in the determination of the antioxidant power of a chemical species. In PE medium, the SET mechanism probably does not contribute to the overall reactivity of higenamine towards ^•^OOH, since such an environment does not provide the necessary solvation of the intermediate ionic species yielded by this mechanism. Accordingly, the calculated ΔG for the SET mechanism were found to be largely endergonic.

The computed rate constants in aqueous solution for all the considered mechanisms are shown in Table 3, which also reports the overall rate coefficient calculated as the sum of the rate constants of each path.

The rate constants showed that for the H_4_A^+^ species, the HAT mechanism was favoured with respect to SET and RAF. In particular, the rate constants for H abstraction from site 3 of catechol were 6.23 and 4.76 times higher than those for site 1 and site 4, respectively. For the H_3_A species, the rate constant associated with the SET mechanism was feasible, with a value equal to 2.64 x 10^8^ L mol^−1^ s^−1^. In general, the results indicated that the rate constant increased in the presence of the anionic form of higenamine.

In order to assert the substantial contribution of each species, the calculated overall rate coefficients were corrected by considering the population of each acid–base form at physiological pH. The derived sum of the corrected-by-fraction total rate coefficients are reported in Table 4.

From Table 4, it is evident that the overall reactivity of higenamine against the ^•^OOH radical is almost entirely due to the presence in aqueous solution of the H_3_A form. Furthermore, even if the total rate coefficient of the anionic form is higher than those of the other two forms, H_2_A^−^ is present in very small quantities and cannot be considered the driving force of the antioxidant activity. On the other hand, at pH 7.4, the most abundant species remains H_4_A^+,^ with a total rate constant of 1.26 × 10^2^ M^−1^ s^−1^.

To put into perspective the potential role of higenamine as an antioxidant, the overall rate coefficient of its reaction with •HOO, in aqueous solution at physiological pH, was compared with those obtained for known antioxidants using a similar methodology. According to the estimated data, higenamine (k_overall_ = 1.23 × 10^8^ M^−1^ s^−1^, this work) is more efficient for scavenging hydroperoxyl radicals than Trolox (8.96 × 10^4^ M^−1^ s^−1^)[47], which is frequently used as a reference antioxidant. It is also more efficient for that purpose than melatonin (2.0 × 10^1^ M^−1^ s^−1^) and caffeine (3.3 × 10^−1^ M^−1^ s^−1^) [48,49], whose protective action against oxidative stress is frequently associated with their ROS scavenging activity [50,51,52].

The results underline that the antioxidant power of higenamine is strongly influenced by pH. Higher pH values increase the protective effects against oxidative stress due to the increase of the H_3_A and H_2_A^−^ forms. In particular, at more basic pHs, SET and SPLET become the favored mechanisms.

## 4. Conclusions

A systematic study of the reactivity of higenamine toward ^•^OOH was carried out in aqueous and lipid environments considering hydrogen transfer, single-electron transfer, and radical adduct formation. In aqueous solution, the species coming from acid–base equilibria were taken into account. It was found that the hydrogen atom transfer from the catecholic ring was the main reaction channel for the H_4_A^+^ species. In contrast, the H_3_A and H_2_A^−^ forms, ^•^OOH scavenging activity took place almost exclusively via SET. Our computed kinetic constants revealed that in water solution it is important to consider all the species derived from acid–base equilibria to obtain reliable results. Comparisons with other species considered good antioxidants revealed that the ^•^OOH scavenging activity of higenamine is higher than that of Trolox. In addition, higenamine resulted to be more efficient than melatonin and caffeine for that purpose.

## Figures and Tables

**Table 1:** Molar fractions (M *f*) of the different acid–base species of higenamine at physiological pH.

H_4_A^+^	H_3_A	H_2_A^−^	HA^2−^	A^3−^
0.861	0.136	0.002	0.000	0.000

**Table 2:** Gibbs free energies of reaction (ΔG) and activation (ΔG^⧧^), expressed in kcal mol^−1^, at 298.15 K. in aqueous solution involved in HAT and SET. The ΔG values calculated in PE medium are reported in parenthesis.

	H_4_A^+^		H_3_A		H_2_A^−^	
	ΔG	ΔG⧧	ΔG	ΔG⧧	ΔG	ΔG⧧
HAT-O4′	1.82	23.35	−0.26	21.80	−3.81	21.36
HAT-O6	−3.62	19.99				
HAT-O7	−2.58	20.93	−13.34	8.49	−15.63	1.33
HAT-C1	2.43		−0.85	13.39		
HAT-C3	16.46		15.58			
HAT-C4	3.19		2.55			
HAT-C7	6.33		7.57			
SET	31.19 (91.53)		4.42 (36.60)		−1.03 (15.11)	
RAF-C7	15.58	23.89				
RAF-C8	19.14	21.61				
RAF-C9	22.73	24.11				
RAF-C10	15.93	20.17				
RAF-C5	19.14	21.61				
RAF-C6	15.65	24.37				
RAF-C1′	19.84	26.42				
RAF-C6′	22.21	28.95				
RAF-C2′	18.17	28.09				
RAF-C5′	18.65	24.36				

**Table 3:** Rate constants (M^−1^ s^−1^) and branching ratios (Γ) computed at the M062x level of theory at 298.15 K.

	H_4_A^+^		H_3_A		H_2_A^−^	
	k	Γ(%)	k	Γ(%)	k	Γ(%)
HAT-O4′	1.62 × 10^1^	11.02	3.11 × 10^1^	~0.00	6.85 × 10^1^	~0.00
HAT-O6	1.01 × 10^2^	68.67	~	~	~	~
HAT-O7	2.12 × 10^1^	14.41	5.03 × 10^8^	65.58	2.79 × 10^9^	29.25
HAT-C1	~	~	1.25 × 10^5^	0.02	~	~
SET	1.04 × 10^−13^	~0.00	2.64 × 10^8^	34.40	6.75 × 10^9^	70.75
RAF-C7	1.53 × 10^−2^	0.01				
RAF-C8	4.07 × 10^−3^	~0.00				
RAF-C9	9.68 × 10^−3^	0.01				
RAF-C10	7.85 × 10^1^	5.34				
RAF-C5	7.22 × 10^−1^	0.49				
RAF-C6	7.15 × 10^−3^	~0.00				
RAF-C1′	2.07 × 10^−4^	~0.00				
RAF-C6′	1.46 × 10^−5^	~0.00				
RAF-C2′	3.43 × 10^−6^	~0.00				
RAF-C5′	5.69 × 10^−2^	0.04				
RAF-C3′	7.37 × 10^−3^	0.01				
RAF-C4′	2.10 × 10^−5^	~0.00				
Overall	1.47 × 10^2^		7.67 × 10^8^		9.54 × 10^9^	

**Table 4:** Molar fractions (𝑓), total rate constants (k_tot_, M^−1^ s^−1^), and corrected-by-fraction total rate coefficients (𝑓k_tot_, M^−1^ s^−1^) at 298.15 K, in aqueous solution at pH 7.4.

	*f*	k_tot_	*f*k_tot_
H_4_A^+^	0.861	1.47 × 10^2^	1.26 × 10^2^
H_3_A	0.136	7.67 × 10^8^	1.04 × 10^8^
H_2_A^−^	0.002	9.54 × 10^9^	1.91 × 10^7^
